# Oridonin targets PRDX1 to promote apoptosis by inducing ROS-mediated ER stress and modulating autophagy

**DOI:** 10.7150/ijbs.110208

**Published:** 2026-06-17

**Authors:** Ziyang Ding, Sisi Wang, Junhui Chen, Yali Song, Zhou Zhu, Haibo Tong, Ashok Iyaswamy, Peng Chen, Xu Wei, Wei Zhang, Jigang Wang, Chuanbin Yang, Yulin Feng

**Affiliations:** 1 School of Pharmaceutical Sciences, National Pharmaceutical Engineering Center for Solid Preparation in Chinese Herbal Medicine, Jiangxi University of Chinese Medicine, Nanchang 330004, China.; 2 Department of Critical Care Medicine, Guangdong Provincial Clinical Research Center for Geriatrics, Shenzhen Clinical Research Center for Geriatrics, Shenzhen People's Hospital (The Second Clinical Medical College, Jinan University, The First Affiliated Hospital, Southern University of Science and Technology), Shenzhen, 518020, Guangdong, China.; 3 Center for Drug Research and Development, Guangdong Provincial Key Laboratory for Research and Evaluation of Pharmaceutical Preparations, Guangdong Pharmaceutical University, Guangzhou, 510006, Guangdong, China.; 4 Department of Reproductive Medicine, Dongguan Maternal and Child Health Care Hospital, Dongguan, 523000, China.; 5 Mr. & Mrs. Ko Chi-Ming Centre for Parkinson's Disease Research, School of Chinese Medicine, Hong Kong Baptist University, Hong Kong SAR, China.; 6 Department of Biochemistry, Karpagam Academy of Higher Education, Coimbatore, India.; 7 Experimental Research Center, China Academy of Traditional Chinese Medicine, Beijing, 100700, China.; 8 Wangjing Hospital, China Academy of Chinese Medical Sciences, Beijing, 100700, China.; 9 State Key Laboratory for Quality Ensurance and Sustainable Use of Dao-di Herbs, Artemisinin Research Center, and Institute of Chinese Materia Medica, China Academy of Chinese Medical Sciences, Beijing, 100700, China.

**Keywords:** oridonin, autophagy, ER stress, oxidative stress, drug targets, chemical proteomics

## Abstract

Oridonin is a bioactive diterpenoid derived from the widely used traditional Chinese medicinal herb *Rabdosia rubescens*, exhibits broad-spectrum anti-cancer activity, with several derivatives currently in clinical trials. However, the molecular mechanism underlying its anticancer effects, especially its direct target proteins, remain to be fully elucidated. Here, we found that Oridonin promoted intracellular reactive oxygen species (ROS) accumulation, which in turn induced endoplasmic reticulum (ER) stress-mediated apoptosis. Moreover, ER stress was instrumental in inducing autophagy after Oridonin treatment, while blockade of autophagy further exacerbated Oridonin-induced cytotoxicity. Notably, using activity-based protein profiling (ABPP), we identified the anti-oxidant enzyme Peroxiredoxin 1 (PRDX1) as a key direct covalent target of Oridonin. By binding to Cysteine 173 of PRDX1, Oridonin increased intracellular ROS levels. Furthermore, PRDX1 over-expression mitigated, whereas PRDX1 knockdown potentiated, Oridonin-induced ROS accumulation, autophagy, and subsequently apoptosis. Overall, our results indicate that PRDX1 is a direct covalent binding target mediating Oridonin-induced apoptosis. These findings not only provide fresh insights into the core mechanism of Oridonin-induced cytotoxicity, but also highlight PRDX1 as a potential therapeutic target for renal cancer drug development.

## Introduction

At present, surgical resection combined with targeted or immunotherapy is the main clinical treatment strategy for multiple malignancies, including renal cell carcinoma (RCC). Beyond surgery, adjuvant and targeted therapies can also be used in patients with metastatic renal cell carcinoma 1. However, conventional chemotherapy drugs often lead to drug resistance and substantial adverse reactions and toxicity, necessitating the development of novel anticancer therapeutic strategies.

In recent years, herbal medicine has been widely explored by clinical and medical researchers for discovering novel drugs, particularly anti-cancer agents 2. *Rabdosia rubescens* for treating cancers, and Oridonin represents its major bioactive compound. Accumulating evidence indicates that Oridonin exhibits robust antitumor activity against diverse malignancies, such as lung, pancreatic, and breast cancers 3. Several derivatives of oridonin are currently undergoing clinical trials for treating cancers 4. Previous studies have reported that Oridonin interacts, either directly or indirectly, with a range of proteins and receptors 5, proteases, transcription factors, and kinases, including telomerase, c-Myc, EGFR, p21, NF-κB, Ras, JNK, and p38 6, 7. However, the underlying mechanism of Oridonin action, particularly its direct protein targets in cancers such as RCC, has not yet been clearly elucidated.

As a by-product of cellular oxidative metabolism, reactive oxygen species (ROS) are essential to both physiological and pathological processes 8, 9. Antioxidant protein content is increased in cancer cells to eliminate ROS and maintain cellular homeostasis. Highly expressed peroxiredoxins (PRDXs), an important family of oxidative stress defense enzyme systems, protect cells against hydroperoxides and are an important mechanism for maintaining critical ROS levels in cancer cells 10, 11. By triggering endoplasmic reticulum (ER) stress, a highly conserved mechanism, increased ROS leads to apoptosis. Notably, under various stresses, ROS can lead to autophagy and/or apoptosis in cancer cells, and autophagy also plays a key role in regulating apoptosis 12. However, whether and how Oridonin modulates these pathways to induce cell death is largely unclear.

The purpose of this study was to elucidate the potential mechanisms of Oridonin-induced cytotoxicity in renal cell carcinoma. We found that Oridonin induced apoptosis in renal cancer cells. Cell death was triggered by accumulation of ROS and subsequent ER stress. Then, we discovered that ER stress is involved in activation of autophagy for survival after Oridonin treatment. Next, protein profiling based on (ABPP) activity identified peroxin-1 (PRDX-1) as a covalent target of Oridonin. In addition, PRDX1 is critical for Oridonin inducing ROS accumulation and apoptosis. Our findings emphasize that PRDX1 is a direct target for Oridonin-induced autophagy and apoptosis in renal cell carcinoma, the mechanism and potential application of Oridonin as an anticancer drug were revealed, which laid a potential foundation for the new progress of cancer therapy.

## Material and Methods

### General reagents

Oridonin, with a purity of 98%, was acquired from Bethealth People Biomedical Technology (Beijing, China). The cell counting kit-8 (CCK-8) was sourced from TargetMol (Shanghai, China). Additionally, the Annexin V apoptosis detection kit I and propidium iodide (PI) were obtained from KeyGEN BioTECH (Nanjing, China). ROS probes, specifically 2', 7'-dichlorodihydrofluorescein diacetate (DCFH-DA), were procured from Aladdin (Shanghai, China), Furthermore, N-acetyl-L-cysteine (NAC) ,4-phenylbutyric acid (4-PBA), Chloroquine (CQ) and Torin1 were purchased from MedChemExpress (New Jersey, USA). The reagents for click chemistry and LC-MS/MS analysis included TAMRA-azide, Biotin-azide, and THPTA, supplied by ClickChemistryTools (Scottsdale, USA). Furthermore, NaVc and CuSO4 originated from Sigma-Aldrich (Missouri, USA). High-capacity neutravidin agarose beads, TEAB, and sequencing-grade trypsin were obtained from Thermo Fisher Scientific (Massachusetts, USA). Antibodies targeting Bcl-2 (15071S), Bax (2772S), Cleaved-caspase 3 (9664T), JNK (9292T) and ATF4 (11815S) were acquired from Cell Signaling Technology (Massachusetts, USA). Antibodies for GRP78 (11587-1-AP), CHOP (15204-1-AP), EIF2α (11233-1-AP), phosphorylated-EIF2α (p-EIF2α, 28740-1-AP), PRDX1 (15816-1-AP) and GAPDH (10494-1-AP) were acquired from Proteintech (Wuhan, China). The P62 antibody (P0067) originated from Sigma-Aldrich (Missouri, USA). The anti-LC3B antibody (NB100-2220S) was sourced from Novus Biologicals (Colorado, USA). HRP-conjugated goat anti-rabbit (A0208) and goat anti-mouse (A0216) immunoglobulins were purchased from Beyotime Biotechnology (Shanghai, China).

### Cultivation of cells

The human renal cell carcinoma (RCC) cell lines 786-O and OSRC-2 (originated from ATCC) were maintained in RPMI 1640 medium (sourced from Gibco, USA), while the normal renal tubular epithelial cell line HK-2 and human embryonic kidney cell line 293T (both obtained from ATCC) were cultured in DMEM (sourced from Gibco, USA).This culture medium was enriched with 10% fetal bovine serum (supplied by Excell, Australia), 100 units/ml penicillin, and 100 μg/mL streptomycin (Gibco, USA).These cells were incubated in a humidified environment containing 5% CO_2_ at a temperature of 37 °C. Prior to drug administration, the cells were allowed to proliferate until they reached confluence. A stock solution of 50 mM was prepared by dissolving Oridonin in dimethyl sulfoxide.

### Assessment of cell viability, colony formation, and apoptosis

786-O, OSRC-2, HK-2, and 293T cells were plated in 96-well plates at a density of 3.5×10^3^ cells/well and exposed to different concentrations of Oridonin for 24-48 h. Cell viability was assessed using CCK-8 assay according to the manufacturer's protocol 13, 14. For colony formation assay, 786-O cells in 6-well plates were treated with Oridonin for 7 days and stained with crystal violet. Apoptosis was evaluated in 6-well plates by treating cells with Oridonin, staining with Annexin V-FITC and PI, and analyzing by flow cytometry. Data were processed using FlowJo v10.

### Proteomic analysis

786-O cells were cultured in a 6-well plate and treated with 15 μM Oridonin or vehicle control for 24 h. Cells were then harvested and proteins were extracted according to our established protocol for proteomics analysis 15, utilizing lysis buffer containing 8M urea, 1% SDC, and 100 mM TEAB in ddH2O. Protein concentrations were then determined using a BCA assay kit. Subsequently, 100 μg of proteins underwent reduction with DTT and alkylation with IAA. Proteins were further precipitated with TCA and digested with Lys-C and trypsin enzymes. The resulting peptides were desalted and analyzed by LC-MS/MS in data-independent acquisition (DIA) mode. For data interpretation, we utilized DIA-NN software (v1.8.0) 16. Differentially expressed proteins (DEPs) were identified based on a fold-change threshold of > 1.2 combined with a p-value < 0.05.

### Measurement of ROS

After being cleaned with PBS and stained with 10 μM DCFH-DA for 30 minutes, the cells were then subjected to flow cytometry analysis.

### Western blotting analysis

786-O and OSRC-2 cells were exposed to various Oridonin concentrations for 24 h. Proteins were extracted using 1% SDS lysis buffer and quantified by BCA assay. SDS-PAGE was utilized to separate equal quantities of protein, followed by their transfer onto PVDF membranes with a pore size of 0.2 μm. Prior to antibody incubation, the membranes were blocked using 5% non-fat milk and then incubated with primary antibodies at 4 °C overnight. The membrane was washed with TBST; membranes were incubated with HRP-conjugated secondary antibody at 4 °C for 2 h, followed by ECL detection 17, 18. Signals were visualized using the G: BOX gel system and analyzed with Image J software.

### *In situ* fluorescence labeling experiments

Activity-Based Protein Profiling (ABPP) involves the identification of proteins based on their functional interactions with chemical probes targeting active compounds, following our previously established protocol 19. In this study, the IAA-yne probe was utilized to assess its competitiveness with Oridonin. Initially, for the purpose of fluorescence labeling within live cells, 786-O cells were plated in a 6-well plate and grown to reach 85%-90% confluence. Then, 786-O cells were pretreated with various concentrations of Oridonin for duration of 2 hours, each followed by the addition of IAA-yne (20 μM) for an additional 1 h. Afterward, the treated cells were collected and lysed using RIPA buffer. Soluble proteins were isolated via centrifugation, and their concentrations were determined using the BCA assay Kit. An equivalent amount of lysate protein (50 μg) from each group was then subjected to a click chemistry reaction cocktail, consisting of NaVc (1 mM), THPTA (100 mM), CuSO_4_ (100 mM) and TAMRA-azide (5 mM). This reaction was conducted with vigorous shaking at RT for 2 h. Following this, the labeled proteins were precipitated using pre-chilled acetone (-20 °C) for 30 minutes. The precipitates were dissolved in 40 μL of 1x loading buffer and loaded onto SDS-PAGE gels for electrophoretic separation. The proteins were visualized through fluorescence scanning using a laser scanner (Azure Sapphire RGBNIR, USA). Ultimately, the SDS-PAGE gels were stained with Instant Blue Coomassie Protein Stain (EpiZyme, Shanghai) for further analysis.

### Pull-down assay and LC-MS/MS analysis

Cells pretreated as outlined in Section 2.7 were utilized. The lysate supernatant underwent a click reaction with NaVc (1 mM), THPTA (100 mM), CuSO_4_ (100 mM) and biotin-azide (50 mM) for 4 h. The reaction mixture was then precipitated in pre-cooled acetone (-20°C) for 1 h and dissolved in PBS containing 1.5% SDS. Overnight incubation at 4 °C was performed with 50 μL of streptavidin beads (Thermo Fisher, Cat. 20349). The beads were subsequently centrifuged, washed with buffers including 1% and 0.1% SDS, 6 M urea, and PBS, and enriched for protein. SDS-PAGE separation and Western blotting detection were conducted. For LC-MS/MS identification of target proteins, the beads-bound proteins were digested with trypsin at 37 °C overnight. Desalting was performed using a C18 column, followed by peptide labeling with TMT 10-plex Mass Tag reagents (Thermo Fisher, Cat. 90113). Finally, the samples were analyzed by LC-MS/MS (Vanquish Flex UHPLC Systems, Thermo Fisher) according to established protocols 20.

### Cell thermal shift assay (CETSA)

CETSA is based on the principle that drug-protein binding increases the thermal stability of target proteins, rendering them less susceptible to heat-induced denaturation, while unbound proteins precipitate and are then removed by centrifugation. Ultimately, the protein present in the supernatant was subsequently detected utilizing Western Blotting, enabling evaluation of drug-target interactions and potential off-target proteins. To substantiate the targeting of proteins by Oridonin, the procedure involved aliquoting the soluble protein lysate derived from 786-O cells into PCR tubes. These aliquots were then subjected to treatment with either Oridonin (200 μM) or DMSO for duration of 2 h at RT, preceding the application of a CETSA. Then, the indicated temperatures, ranging from 37 °C to 57 °C, were applied to the solutions for 3 minutes in an Applied Biosystems (USA) thermocycler. Subsequently, the solutions were centrifuged at 20000xg and 4°C for 20 minutes. The resulting soluble supernatant was then analyzed by Western blotting.

### Production and purification of human recombinant proteins

Human PRDX1 (GenBank ID: NP_859048.1) and Cys52Ser (Sangon, China) mutant were cloned into pET28a vector as previously described 21. The recombinant plasmids were transformed into BL21 cells, which were cultured in LB containing kanamycin until the optical density at 600 nm (OD600) reached 0.8-1.2. Protein expression was induced with isopropyl β-D-1-thiogalactopyranoside (IPTG), and incubated at 16 °C for 14 h. Cells were harvested, lysed, centrifuged, and the supernatant was loaded onto Ni-NTA affinity column (Qiagen, USA). Bound proteins were eluted with imidazole-containing buffer; and protein concentrations were measured using a BCA kit. Protein purity and integrity were assessed by SDS-PAGE followed by Coomassie brilliant blue staining.

### Molecular docking simulation

The PRDX1 structure (PDB ID: 4XCS) was retrieved from the Protein Date Bank (PDB). Hydrogenation and dehydration processes were carried out using discovery studio client software. Docking simulations and mapping were subsequently performed using PyMOL and Pyrx-0.8 software, respectively.

### Plasmid construction and cell transfection

Cells was transfected with ployethylenimine with a linear molecular weight of 25000 (PEI 25K, 23966-1) as described previously 22, 23. Specifically, the plasmids RFP-GFP-LC3 and GFP-LC3 were utilized to evaluate autophagic flux and autophagosome formation, respectively. After treating the transfected cells with specified compounds for 24 h, they were fixed with 4% PFA. Cellular images were captured using a Nikon ECLIPSE Ti2 microscope and analyzed with Image J software to count the number of GFP-LC3 puncta per cell.

### Activity assay of recombinant human PRDX1 protein

The peroxidase activity of recombinant human PRDX1 (rhPRDX1) was assessed via H₂O₂ reduction according to previous report 20. Briefly, serially diluted rhPRDX1 was reacted with 50 μM H₂O₂ (37 °C, 15 min), and residual H₂O₂ was quantified using a commercial assay kit (Beyotime, China) by measuring absorbance at 560 nm. For inhibition assays, rhPRDX1 (30 μM) was pre-incubated with Oridonin or vehicle control (25 °C, 20 min) prior to activity measurement. Relative activity was normalized to DMSO-treated controls.

### Animal experiments

Five-week-old male nude mice (BALB/c background, 20 ± 2 g) were obtained from GemPharmatech (Guangzhou, China). Mice were housed under specific pathogen-free conditions with controlled temperature (22 ± 1 °C) and 12 h light/dark cycles. All experimental protocols were approved by the Animal Ethics Committee of Shenzhen People's Hospital (Shenzhen, China). Subcutaneous xenografts were established by injecting 5 × 10^6^ OSRC-2 renal carcinoma cells (resuspended in 100 μL PBS) into the right flank. Beginning 10 days post-inoculation, mice received intraperitoneal injections of 5 mg/kg Oridonin and 50 mg/kg hydroxychloroquine (HCQ, dissolved in 30% PEG300, 5% DMSO, and 65% sterile water) or vehicle control (30% PEG300/5% DMSO/65% water) every 3 days for 3 weeks. Tumor dimensions were measured every 3 days using digital calipers, and volumes were calculated as: Volume = 0.5 × length × width^2^.

### Statistical analysis

The results are reported as mean ± SEM. Multiple-group experimental data were analyzed via one-way ANOVA with Tukey's post-hoc test. For pairwise comparisons, an unpaired, two-tailed t-test was applied. GraphPad Prism software (v8.0) was utilized for all statistical assessments. Levels of significance are indicated by asterisks, where * signifies *P* < 0.05, ** signifies* P* < 0.01, and *** signifies *P* < 0.001.

## Results

### Oridonin inhibits cells proliferation and induces apoptosis

**Figure 1:**
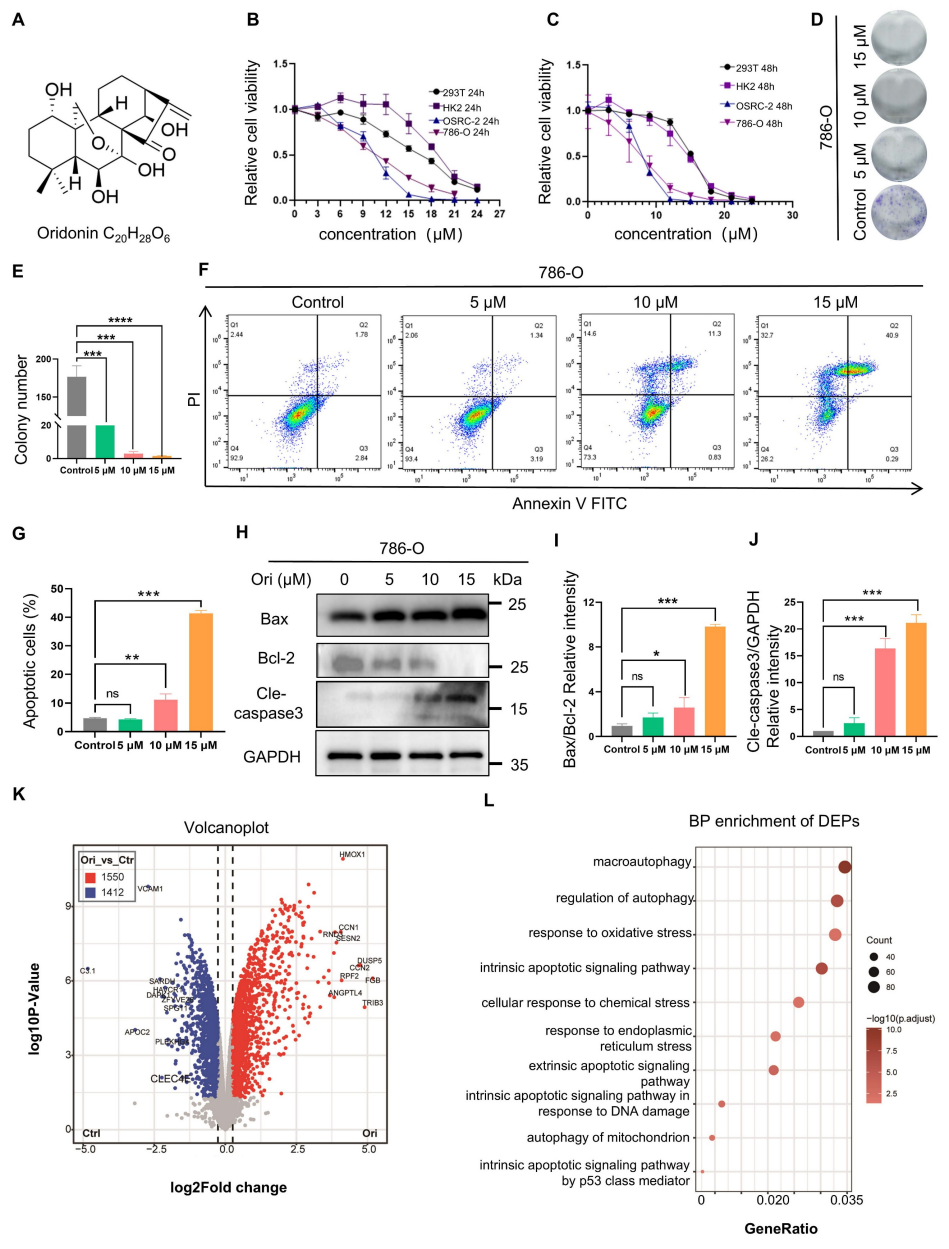
**Oridonin inhibits proliferation and induces apoptosis in RCC cells.** (A) Chemical structural formula of the compound Oridonin (Ori). (B-C) CCK-8 assay to detect the effect of Oridonin on the viability of various cells. Human embryonic kidney epithelial cells (293T), normal renal tubular epithelial cells (HK-2), and RCC cell lines (786-O, OSRC-2) were exposed to different concentrations of Oridonin (0-24 μM) and treated for 24 or 48 h, respectively. (D-E) Colony formation assay. After treating 786-O cells with Oridonin (10-15 μM) for 7 days, the colony formation rate of the cells decreased in a dose-dependent manner. (F-G) Oridonin induces apoptosis. After 24 h of treatment with Oridonin (10-15 μM), apoptosis of 786-O cells was assessed by flow cytometry and then quantitative analysis. (H-J) Western blot analysis of apoptosis-related protein expression: 24 h after Oridonin (10-15 μM) treatment, Western blotting was performed to detect the protein expression of Bax, Bcl-2, and cleaved caspase-3 in Oridonin-treated 786-O cells Bax, Bcl-2 and cleaved caspase-3 in 786-O cells treated with oridonin. (K) Volcano plot of proteomics results highlighting 2962 differential expressed proteins, with 1550 up-regulated and 1412 down-regulated after Oridonin treated. (L) GO analysis (focused on biological processes) of differentially expressed genes (DEGs) following Oridonin treatment. The presented data are expressed as mean ± SEM, derived from a minimum of three separate experiments, with statistical significance assessed through comparison to the control group (*P < 0.05, **P < 0.01, ***P < 0.001).

First, we investigated the effects of oridonin on cell viability in normal cell lines and renal cancer cell lines, respectively. As determined by CCK-8 assay, Oridonin exhibited dose-dependent inhibitory effects on cell viability in both renal cancer and normal cell lines. In renal carcinoma cells, the IC50 values decreased from 10.6 μM (24 h) to 7.75 μM (48 h) for 786-O, and from 10.53 μM (24 h) to 8.1 μM (48 h) for OSRC-2. In contrast, normal renal cells showed higher resistance: HK-2 cells displayed IC50 values of 18.8 μM (24 h) and 14.3 μM (48 h), while 293T cells had IC50 values of 16.65 μM (24 h) and 15.1 μM (48 h) (**Fig. 1B-C**). These results demonstrate that Oridonin preferentially targets renal cancer cells (786-O and OSRC-2) over normal renal cells (HK-2 and 293T), with enhanced potency upon prolonged exposure, suggesting potential therapeutic selectivity. Furthermore, the colony formation experiments showed that the growth of 786-O decreased with increasing Oridonin concentration (**Fig. 1D-E**), suggesting that Oridonin significantly inhibits the proliferation of 786-O. In addition to inhibiting proliferation, Oridonin also significantly increased the apoptosis ratio (**Fig. 1F-G**). Additionally, further detection of apoptosis-related proteins (**Figure 1H-J**) revealed significantly elevated Bax/Bcl-2 ratios and cleaved caspase-3 (Cle-caspase3) levels in cells treated with Oridonin. These results suggest that Oridonin induces apoptosis in 786-O cells.

Next, to gain deeper insights into the potential mechanism underlying the cytotoxicity induced by Oridonin, proteomic profiling was conducted on 786-O cells that had been treated with Oridonin. For this analysis, three replicates per group were used to ensure statistical significance (**Fig. S1A**). Initially, we observed a significant divergence in gene expression distance between the vehicle group and the Oridonin-treated cell group (**Fig. S1B-C**). Further investigation revealed the expression patterns of numerous differential expression proteins (DEPs) in the Oridonin-treated cells, which provided additional insights into the mechanism of cytotoxicity (**Figs. S1D**).Through the volcanic map, we found that 2962 differential proteins were detected, of which 1550 were up-regulation and 1412 were down-regulation (Fig. 1I, and Table S1).Using these DEPs for KEGG analysis, we discovered that the differential proteins were significantly enriched in autophagy, oxidative stress, endoplasmic reticulum stress and endogenous apoptosis-related pathways (**Fig. 1L, red box**). These results indicate that these pathways may critical for Oridonin-induced cell death.

### ROS accumulation is critical for Oridonin-induced cell apoptosis

**Figure 2:**
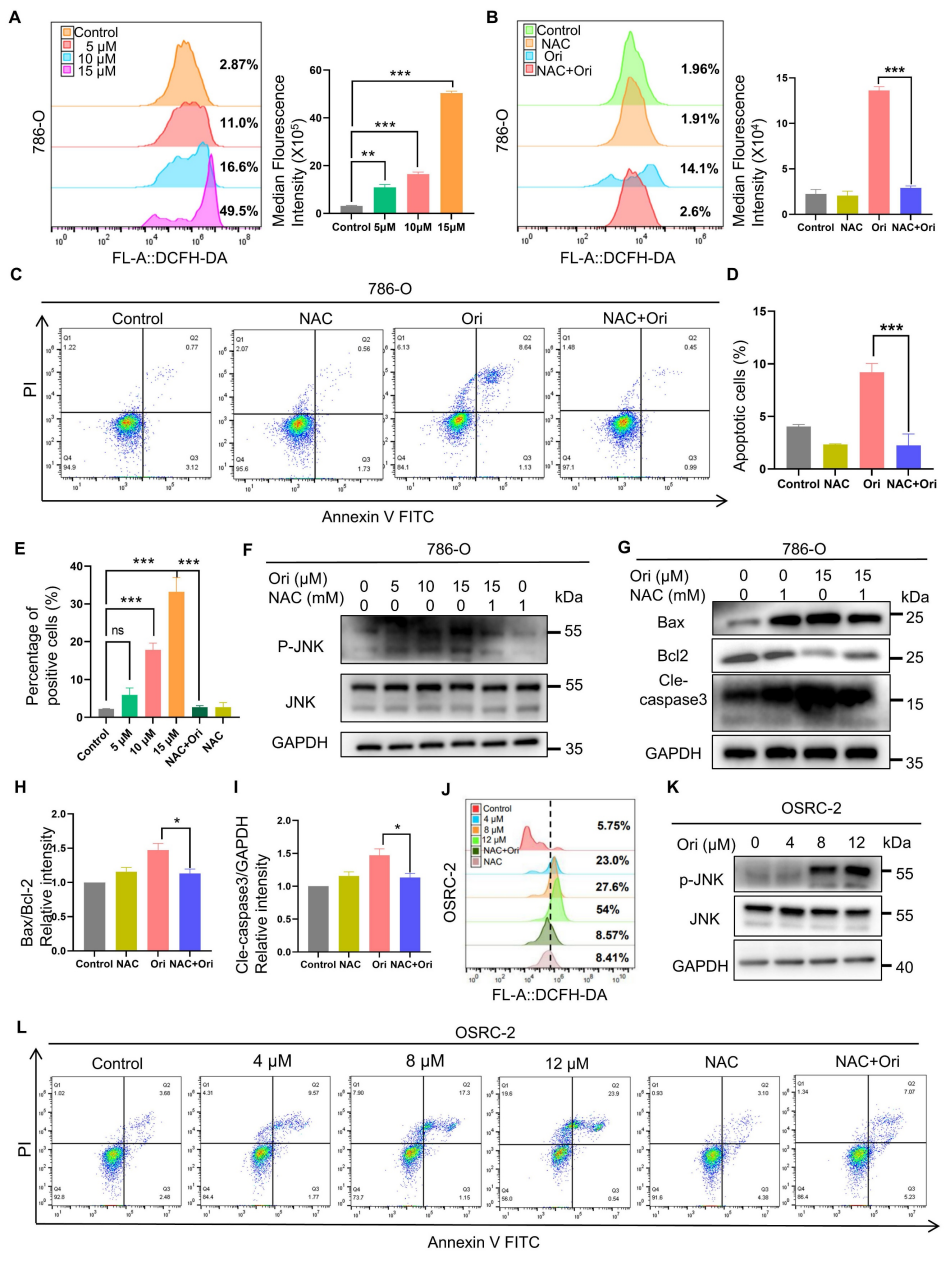
**ROS accumulation contributes to Oridonin-induced cell apoptosis.** (A-B) Oridonin induced the accumulation of reactive oxygen species (ROS) in 786-O cells. Intracellular ROS levels were assessed and quantified using DCFH-DA staining after exposing 786-O cells to oridonin (5-15 μM) for 24 h or co-treatment of oridonin (15 μM) and NAC (1 mM) for 24 h. The ROS levels were quantified using DCFH-DA staining. (C-D) NAC inhibits Oridonin-induced apoptosis. 786-O cells were exposed to Oridonin (15 μM) co-treated with NAC (1 mM) for 24 h. Apoptosis was assessed by flow cytometry. (E) Mitochondrial membrane potential assay. After exposing 786-O cells to oridonin (5-15 μM) for 24 h, they were incubated with JC-1 staining solution for 20 min at 37°C, protected from light, and flow cytometry analysis was performed to detect mitochondrial membrane potential. (F-I) Western blot analysis was conducted to detect the expression levels of P-JNK, JNK (F), Bax, Bcl-2, and cleaved caspase-3 (G) in 786-O cells treated with Oridonin and NAC (1 mM). The presented data are expressed as mean ± SEM, derived from a minimum of three separate experiments, with statistical significance assessed through comparison to the control group (*P < 0.05, **P < 0.01, ***P < 0.001). (J) Oridonin induces the accumulation of reactive oxygen species (ROS) in OSRC-2 cells. After exposing OSRC-2 cells to different concentrations of oridonin and NAC for 24 h, intracellular ROS levels were assessed using DCFH-DA staining followed by flow cytometry analysis. (K) Western blot analysis was performed to detect the expression levels of p-JNK and JNK in OSRC-2 cells treated with different concentrations of oridonin. (L) Apoptosis of OSRC-2 cells was assessed by flow cytometry after exposure to different concentrations of oridonin and the NAC for 24 h.

**Figure 3:**
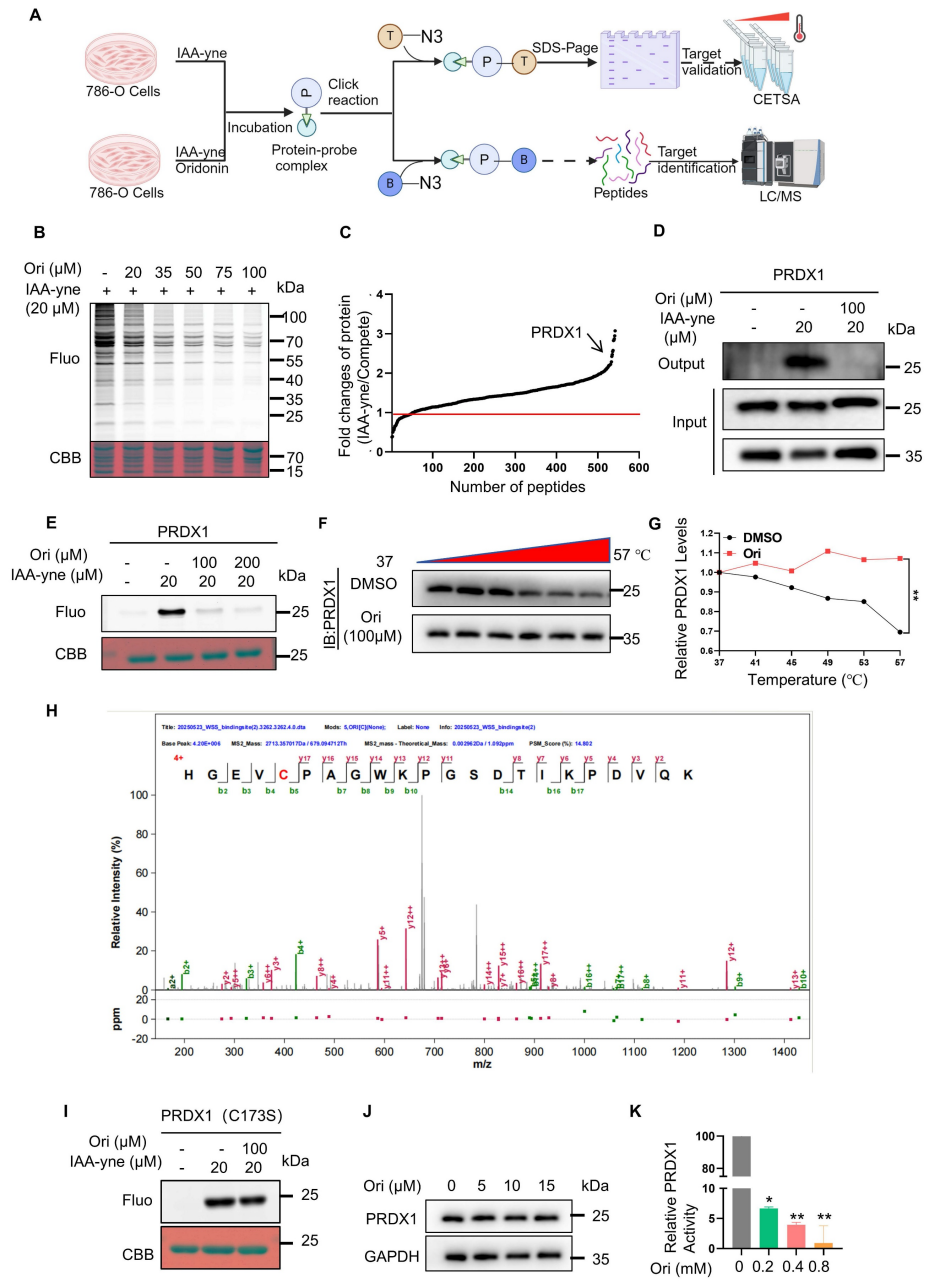
** Oridonin directly binds to the anti-oxidant enzyme PRDX1.** (A) A workflow is outlined for profiling potential protein targets of Oridonin using ABPP. (B) Binding of Oridonin to cysteine residues of proteins occurs competitively with IAA-yne. (C) Chemical proteomics is employed to identify potential binding targets of Oridonin in 786-O cells. (D) Binding of Oridonin to PRDX1 is validated through a pull-down assay. (E) Competitive binding between Oridonin and IAA-yne is observed for purified recombinant human PRDX1 protein. (F-G) CETSA-WB experiments reveal that PRDX1 exhibits significant thermal stability in the presence of Oridonin. (H) Characterization of binding sites of Oridonin on purified recombinant PRDX1 protein (Cysteine 173). (I) In vitro studies show that Oridonin cannot compete with IAA-yne for binding to a point-mutated form of human recombinant PRDX1 (C173S). (J) Oridonin did not significantly inhibits the expression of PRDX1 after treatment of 786-O cells for 24h. (K) Peroxidase activity assay showed that Oridonin inhibits PRDX1's activity.

To further examine whether oxidative stress is involved in Oridonin-induced cell death as suggested by our proteomics results, we first measured ROS levels using DCFH-DA probe staining. Our results showed that Oridonin treatment dose-dependently increased ROS production in 786-O cells (**Fig. 2A**). Furthermore, ROS inhibitor N-Acetyl Cysteine (NAC) mitigated the elevation of ROS levels induced by Oridonin treatment in 786-O cells as expected (**Fig. 2B**). Meanwhile, NAC also significantly restored cell viability and apoptosis induced by Oridonin (**Fig. S2A, Fig. 2C-D**). These results indicate that Oridonin-induced apoptosis via ROS accumulation. Previous report showed that ROS acts upstream of mitochondrial depolarization to induce apoptosis 19. We determined whether mitochondrial apoptotic pathway mediates Oridonin-induced apoptosis. The result showed that Oridonin induced mitochondrial membrane potential (MMP)-depolarized in 786-O cells as revealed by JC-1 staining. Moreover, NAC significantly restore the MMP induced by Oridonin (**Fig. 2E, Fig. S2B**). JNK is a member of the mitogen-activated protein kinase (MAPK) family and plays a pivotal role in cellular stress responses, including oxidative stress, inflammation, and apoptosis. Its activation is critical to cell survival, proliferation, and death 24, we next investigated whether JNK is involved in Oridonin-induced cell toxicity. The levels of p-JNK were significantly elevated in 786-O cells and OSRC-2 cells after treatment with oridonin (**Fig. 3F, K**), whereas inhibition of NAC eliminated oridonin-induced p-JNK suggesting that JNK, a downstream of ROS, may play a role for Oridonin-induced apoptosis. Moreover, NAC significantly restored Oridonin-induced the changes of apoptosis-associated markers Bax, and Cleaved-caspase 3, and increased Bcl-2 levels (**Fig. 2G-I**). Similarly, In OSRC-2 cells, ROS production increased dose-dependently after oridonin treatment, and the ROS inhibitor NAC attenuated the increase in ROS levels induced by oridonin treatment (**Fig. 2J**) and oridonin-induced apoptosis in OSRC-2 cells (**Fig. 3L**). Collectively, these results suggest that Oridonin-induced ROS accumulation activates the mitochondrial apoptotic pathway.

### Oridonin directly binds to the anti-oxidant enzyme PRDX1

To understand how Oridonin induces ROS accumulation and subsequently cell death, we aimed to identify its potential targets (**Fig. 3A**). Oridonin's d-ring α-methylene cyclopentanone acts as a Michael acceptor, which can covalently bind to cysteine sulfhydryl groups in proteins 21. To take full advantage of this characteristic of Oridonin, the well-established ABPP method was used to identify its protein targets. Our results showed that Oridonin competed with the cysteine-reactive probe IAA-yne for protein binding in 786-O cells (**Fig. 3B**). Subsequently, protein samples subjected to treatment with IAA-yne and Oridonin underwent LC-MS/MS analysis to identify potential protein targets capable of covalent binding to Oridonin. Notably, among the identified proteins, PRDX1 exhibited a marked alteration (**Fig. 3C**), drawing our attention because of its anti-oxidant property and involvement in cell death. In addition, using a pull-down experiment, we found that Oridonin effectively reduced the enrichment of PRDX1 protein mediated by IAA-yne probe (**Fig. 3D**). Further gel-based fluorescence analysis revealed that Oridonin substantially diminished the fluorescent labeling of recombinant human PRDX1 protein by the IAA-yne probe (**Fig. 3E**), reinforcing the notion of its covalent binding to cysteine residues within the protein. Additionally, through the application of CETSA, PRDX1 exhibited notable thermal stability when exposed to Oridonin (**Fig. 3F-G**), highlighting PRDX1 is a direct interaction partner of Oridonin. To delve deeper, we incubated recombinant human PRDX1 protein with Oridonin followed by LC-MS/MS. The MS analysis showed that the Cys 173-containing peptide had a mass shift (**Fig. 3H**), suggesting that the Cys 173 of PRDX1 was covalent modified by Oridonin. Consistently, in contrast to WT protein, competitive binding assays revealed that Oridonin failed to compete with the alkylation probe IAA-yne for binding to purified recombinant human Cys173 mutated recombinant PRDX1 (**Fig. 3I**). In addition, we tested whether oridonin affects the expression of PRDX1 and whether binding to PRDX1 affects its function by PRDX1 peroxidase activity assay. The results showed that oridonin did not significantly inhibit the expression of PRDX1 (**Fig. 3J**), but inhibited its enzyme activity (**Fig. 3K**), indicating that oridonin binds to and inhibits the activity of PRDX1. Taken together, these results underscore that Oridonin covalently binds and inhibits its activity, potentially modulating the protective effects of PRDX1 against oxidation and apoptosis in renal cancer cells.

### Oridonin activates apoptotic via ROS-mediated endoplasmic reticulum (ER) stress

**Figure 4:**
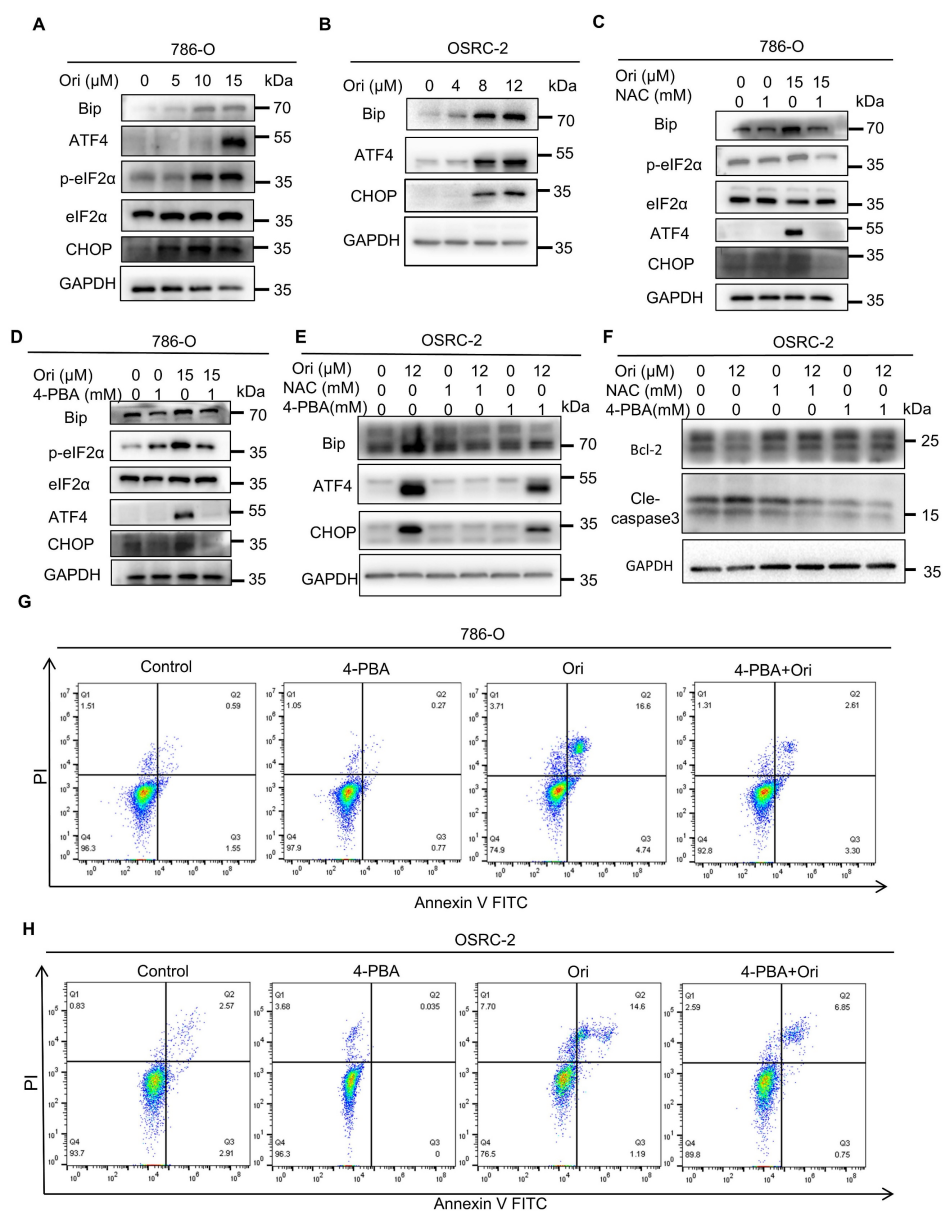
** Oridonin activates apoptotic cell death via ROS-mediated endoplasmic reticulum (ER) stress.** (A-B) Oridonin activates the ER stress pathway in 786-O and OSRC-2 cells. Western blot analysis was used to detect ER stress marker-related proteins in 786-O and OSRC-2 cells treated with different concentrations of oridonin. (C-E) Pre-treatment with the ROS inhibitor NAC (1 mM) mitigates the Oridonin-induced up-regulation of ER stress-related markers in 786-O and OSRC-2 cells. The ER stress inhibitor 4-PBA (1mM) prevented the Oridonin-induced increase in ER stress markers in 786-O and OSRC-2 cells. (F) Western blot analysis to detect the protein expression levels of Bcl-2 and cleaved caspase-3 in OSRC-2 cells after co-treatment of oridonin with NAC (1 mM) or 4-PBA (1 mM). (G-H) Pre-treatment using the ER stress inhibitor 4-PBA(1mM) attenuated Oridonin-triggered apoptosis. The occurrence of apoptosis was identified through Annexin V-FITC/PI staining, followed by flow cytometry analysis. All experiments with Oridonin, presented as mean ±SEM, are based on at least three independent replicates. Statistical significance was determined by comparison with the control group, with asterisks denoting levels of significance (*P<0.05, **P<0.01, ***P<0.001).

Because our proteomics results showed that ER stress is enriched in Oridonin-treated cells, and Oridonin induces ROS accumulation, we next asked whether ER stress is involved in Oridonin-induced cell death. To validate our hypothesis, we initially evaluated multiple key proteins in the endoplasmic reticulum (ER) stress pathway, including GRP78/Bip, P-EIF2α, ATF4, and CHOP. The results showed that the expression levels of these proteins were significantly upregulated, indicating that Oridonin-induced activation of ER stress plays a crucial role in inducing apoptosis in 786-O and OSRC-2 cells (**Fig. 4A-B**). To investigate the role of ROS in ER stress induction, we treated cells with ROS scavenger NAC, which reversed Oridonin-induced upregulation of ER stress proteins (**Fig. 4C, E**). Furthermore, treatment with 4-PBA, an ER stress inhibitor, not only mitigated the increase in ER stress markers in 786-O and OSRC-2 cells (**Fig. 4D-E**), but also effectively decreased Oridonin-induced apoptosis (**Fig. 4F-H**). In addition, we further explored PRDX1's downstream targets, JNK activation, and their contribution to apoptosis. We tested whether inhibition of JNK signaling using the JNK inhibitor SP600125 could attenuate Oridonin-induced downstream signaling activation, ROS accumulation, ER stress activation and subsequent cell death. The results showed that the JNK inhibitor SP600125 was effective in restoring Oridonin-induced ROS accumulation (**Fig. S4E**), ER stress activation and apoptosis (**Fig. S4C-D, F**). These results suggest that inhibition of JNK activation by PRDX1 inhibition may play a critical role in Oridonin-induced apoptosis. These results collectively show ROS accumulation induces ER stress, which subsequently induces apoptosis in Oridonin-treated cells.

### Oridonin-induced ER stress induces cytoprotective autophagy

**Figure 5:**
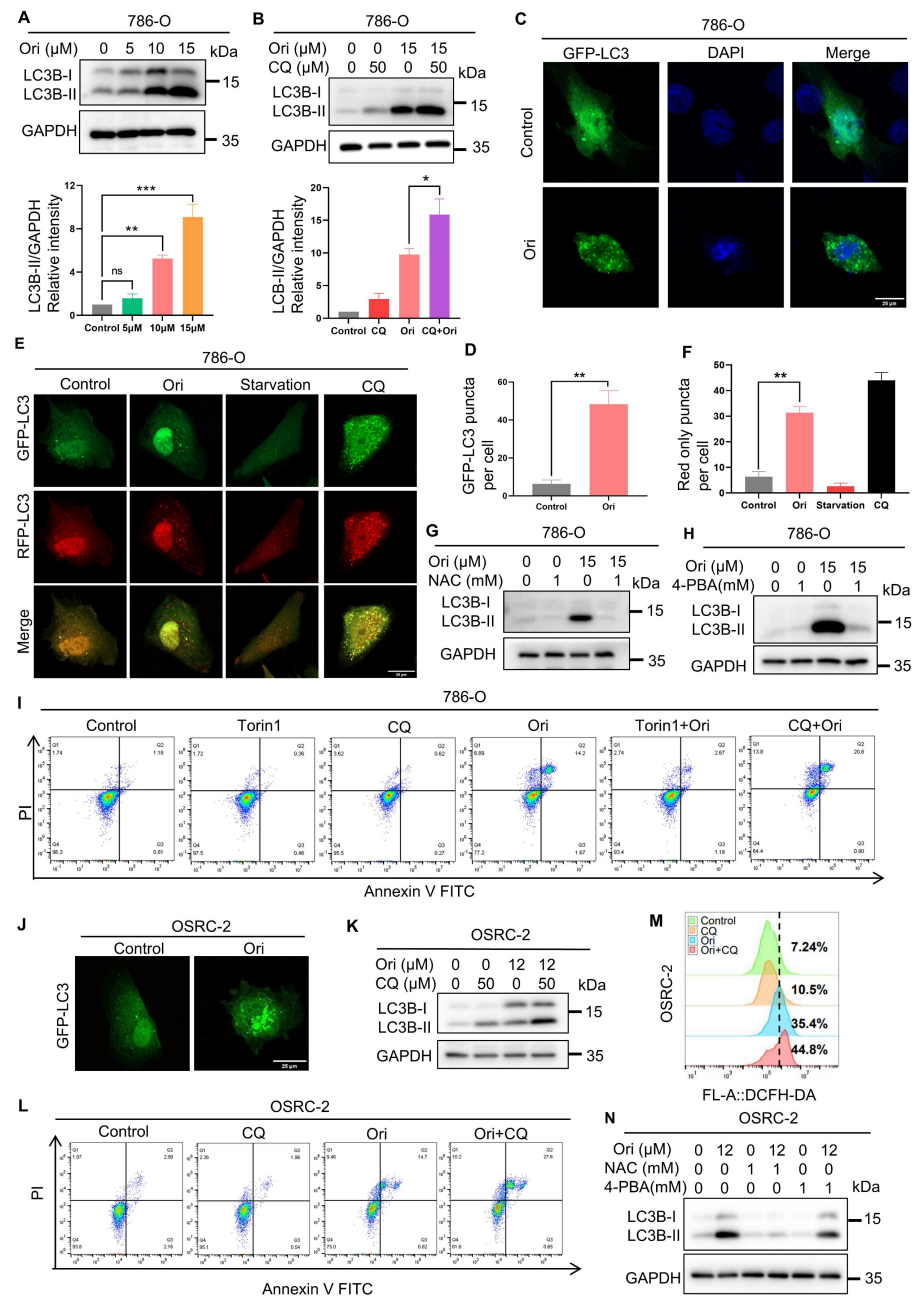
**Oridonin-induced ER stress-mediated cytoprotective autophagy.** (A) Oridonin stimulates the expression of the autophagy marker LC3-II in 786-O cells. Western blotting was employed to detect the relative abundance of LC3-II in cells exposed to specified concentrations of Oridonin for 24 h. Quantification of LC3-II expression levels was performed. (B) Oridonin-induces autophagy flux. Cells were treated with lysosome inhibitor CQ (50 μM) alone or combined with Oridonin, followed by western blotting to measure LC3-II expression. (C-D) Oridonin increased GFP-LC3 puncta in 786-O cells. Cells transiently expressed GFP-LC3 and then treated with Oridonin for 24 h. Images were acquired using fluorescence microscopy and then quantitatively analyzed. (E-F) Oridonin increased red dot-like aggregation in 786-O cells expressing RFP-GFP-LC3. Cells transiently expressing RFP-GFP-LC3 were then treated with oridonin (15 μM) or CQ (50 μM) or starvation for 24 h. Images were acquired using fluorescence microscopy and quantitatively analyzed. (G) The ROS inhibitor NAC (1 mM) attenuates Oridonin-induced autophagy in 786-O cells, and LC3B-II protein expression was analyzed by Western blotting after 24 h treatment with NAC (1 mM) alone or in combination with Oridonin. (H) The ER stress inhibitor 4-PBA (1 mM) reduces Oridonin-induced autophagy in 786-O cells, and LC3B-II protein expression was analyzed by Western blotting after 24 h treatment with 4-PBA (1 mM) alone or in combination with Oridonin. (I) The autophagy activator Torin1 (200 nM) and the lysosomal inhibitor CQ (50 μM) regulated the percentage of apoptosis in oridonin-treated 786-O cells. Apoptosis was detected by Annexin V-FITC/PI staining and flow cytometry. (J) Oridonin increased GFP-LC3 puncta in OSRC-2 cells. Cells transiently expressing GFP-LC3 were treated with oridonin for 24 h and images were acquired using fluorescence microscopy. (K) Oridonin induces autophagy flux. OSRC-2 cells were treated with the lysosomal inhibitor CQ alone or in combination with Oridonin, and then LC3-II expression was determined by Western blotting. (L) The lysosomal inhibitor CQ (50 μM) regulates the percentage of apoptosis in Oridonin-treated OSRC-2 cells, as analyzed by Annexin V-FITC/PI staining and flow cytometry detection. (M) Autophagy attenuates oridonin-induced ROS accumulation. OSRC-2 cells were co-treated with lysosomal inhibitor CQ (50UM) and oridonin, and then intracellular ROS levels were detected by flow cytometry. (N) Cells were co-treated with the ROS inhibitor NAC (1 mM) or the ER stress inhibitor 4-PBA (1 mM) and Oridonin, and LC3B protein levels were detected by Western blotting. Data, presented as mean ± SEM, represent results from at least three independent experiments. Statistical significance (*P < 0.05, **P < 0.01, ***P < 0.001) was compared to the control group.

Previous research has demonstrated that ER stress serves as a key inducer of autophagy, a mechanism vital for modulating cell death pathways 25. Our proteomic analysis revealed autophagy in Oridonin-treated cells. We investigated Oridonin's regulatory effects on autophagy and found a dose-dependent increase in LC3-II levels in Oridonin-treated 786-O cells (**Fig. 5A**). Lysosomal inhibitor chloroquine (CQ) further elevated LC3-II levels (**Fig. 5B, K**), indicating Oridonin facilitates autophagosome formation. Immunofluorescence analysis confirmed Oridonin increased the formation of autophagosomes (**Fig. 5C-D, J**). Next, using the mRFP-GFP-LC3 plasmid, we observed autophagy flux and found that Oridonin treatment significantly increased red-only puncta, indicating its role in augmenting autophagic flux (**Fig. 5E-F**). To examine the function of autophagy in cell death induced by oridonin, we administered the autophagy activator Torin1 and the autophagy suppressor CQ to the cells. Torin 1 enhanced further increased LC3-II levels (**Fig. S3A**), and attenuated apoptosis induced by Oridonin, underscoring autophagy's protective role in Oridonin-induced apoptosis. Conversely, CQ treatment increased the apoptosis ratio (**Fig. 5I, L**), implying that autophagic flux inhibits further promote apoptosis in Oridonin-treated cells.

Finally, we analyzed the interplay between autophagy and ER stress induced by ROS and mediated through Oridonin. Simultaneous use of NAC, an inhibitor of ROS, reversed the elevated LC3-II levels (**Fig. 5G, N**), indicating ROS is involved in Oridonin-induced autophagy. Similarly, the ER stress inhibitor 4-PBA inhibited the increase in LC3-II (**Fig. 5H, N and Fig. S3B**), suggesting a role for ER stress in this process. In addition, Oridonin treatment increased intracellular ROS levels in OSRC-2 cells, and co-treatment with the autophagy inhibitor CQ further increased ROS accumulation, suggesting that autophagy may attenuates oxidative stress (**Fig. 5M**). Collectively, Oridonin induces cell death via ROS-mediated ER stress, which activates cytoprotective autophagy.

### PRDX1 is critical for Oridonin-induced ROS accumulation and subsequently cell apoptosis

**Figure 6:**
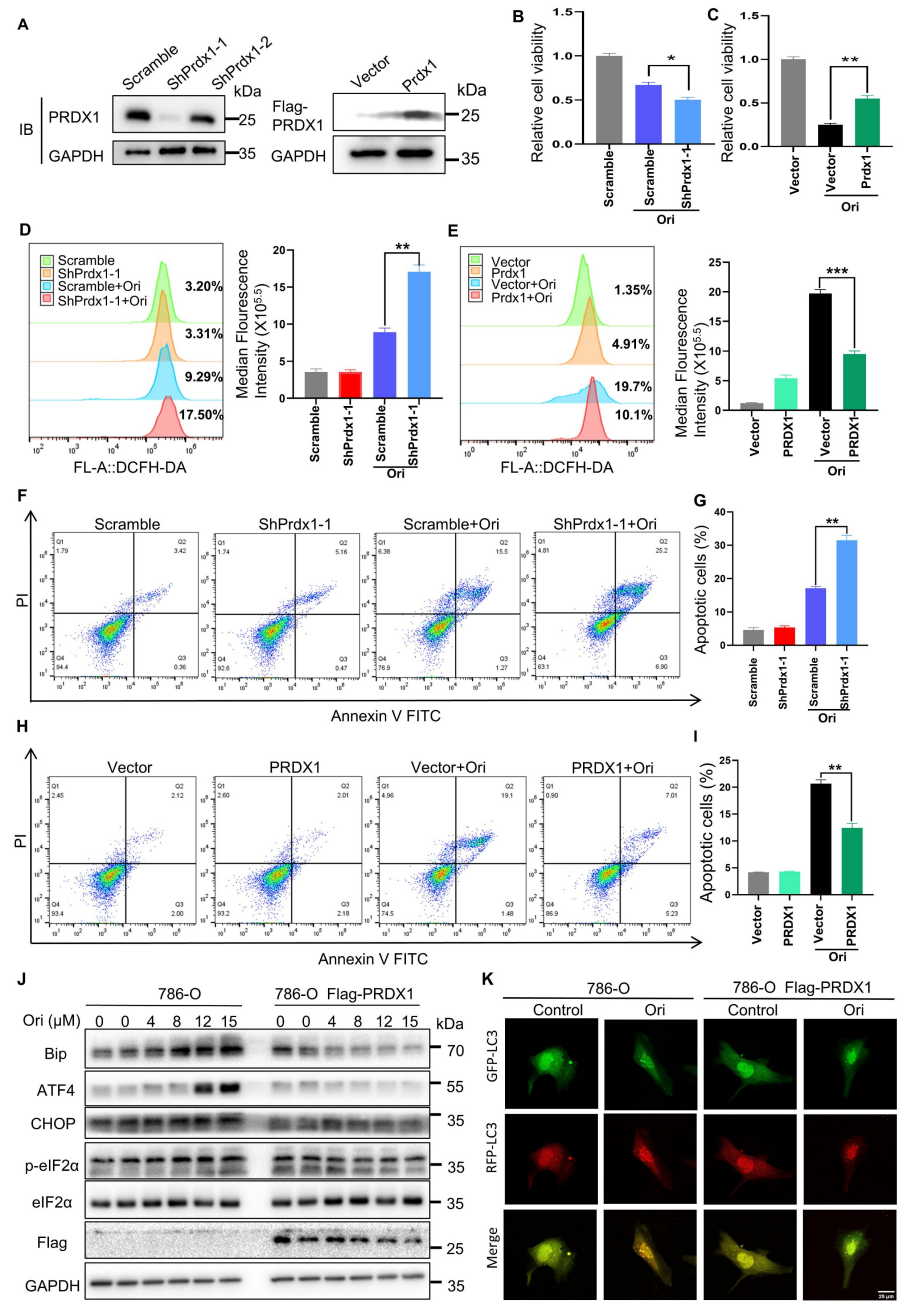
** PRDX1 is critical for oridonin-induced apoptosis**. (A) PRDX1 expression in 786-O cells was modulated via shRNA-induced knockdown (yielding shPRDX1-1 and shPRDX1-2 lines) and overexpression (OE PRDX1 lines). Western blotting assessed PRDX1 levels in cells transfected with PRDX1-targeted shRNA vectors (scramble control) or overexpressing vectors (vector control). (B-C) PRDX1 knockdown enhanced Oridonin's inhibitory effect on 786-O cell viability (B), whereas overexpression attenuated this effect (C). (D-E) PRDX1 knockdown increased ROS accumulation in Oridonin-treated 786-O cells (D), whereas overexpression decreased ROS under the same conditions (E). ROS levels were quantified using DCFH-DA. (F-G) PRDX1 knockdown elevated the apoptosis rate in Oridonin-treated cells, detected by Annexin V-FITC/PI staining and flow cytometry (F), with quantification in (G). (H-I) PRDX1 overexpression reduced the apoptosis rate under Oridonin treatment, detected by Annexin V-FITC/PI staining and flow cytometry (H), with quantification in (I). (J) ER stress marker proteins GRP78, ATF4, CHOP, and phosphorylated eIF2α protein expression levels in 786-O versus PRDX1 overexpressing (OE) 786-O cells were detected by protein immunoblotting assay. (K) RFP-GFP-LC3 was transiently expressed in 786-O with PRDX1 overexpression (OE) in 786-O cells treated with oridonin for 24 h. Images were acquired using fluorescence microscopy. The presented data are expressed as mean ± SEM, derived from a minimum of three separate experiments, with statistical significance assessed through comparison to the control group (*P < 0.05, **P < 0.01, ***P < 0.001).

PRDX1 is recognized as an anti-oxidant enzyme that scavenges hydrogen peroxide and alkyl hydroperoxides 26, playing a pivotal role in reducing ROS production and promoting anti-apoptotic effects in cancer cells. To validate the impact of PRDX1 on Oridonin-induced cell death, we manipulated PRDX1 expression levels in 786-O cells by knocking down (KD) and overexpressing (OE) it using a Flag-tagged PRDX1 (**Fig. 6A**). Notably, our findings revealed that PRDX1 KD cells exhibited increased sensitivity to Oridonin-induced reduction in cell viability compared to scrambled control cells (**Fig. 6B**). Conversely, overexpression of PRDX1 protected cells from Oridonin-induced reduction in viability (**Fig. 6C**). Importantly, PRDX1 knockdown significantly augmented, whereas PRDX1 overexpression markedly suppressed, Oridonin-induced ROS generation (**Fig. 6D-E**) and apoptosis (**Fig. 6F-I**). Importantly, Overexpression of PRDX1 (786-O cells stably expressing Flag-Prdx1) effectively rescued Oridonin-induced the upregulation of ER stress marker proteins such as GRP78, CHOP, ATF4, and phosphorylated eIF2α as detected by protein immunoblotting experiments (**Fig. 6J**). We also found that PRDX1 overexpression inhibited Oridonin-induced autophagy flux in cells expressing mRFP-GFP-LC3 plasmid (**Fig. 6K**). These results further support the critical role of PRDX1 inhibition in Oridonin-induced ROS accumulation, ER stress, autophagy and subsequently cell death. These observations robustly support the hypothesis that Oridonin triggers ROS-mediated apoptosis through covalent binding to PRDX1.

### Combination of oridonin and autophagy inhibitor hydroxychloroquine (HCQ) synergistically suppresses renal cell carcinoma growth *in vivo*

**Figure 7:**
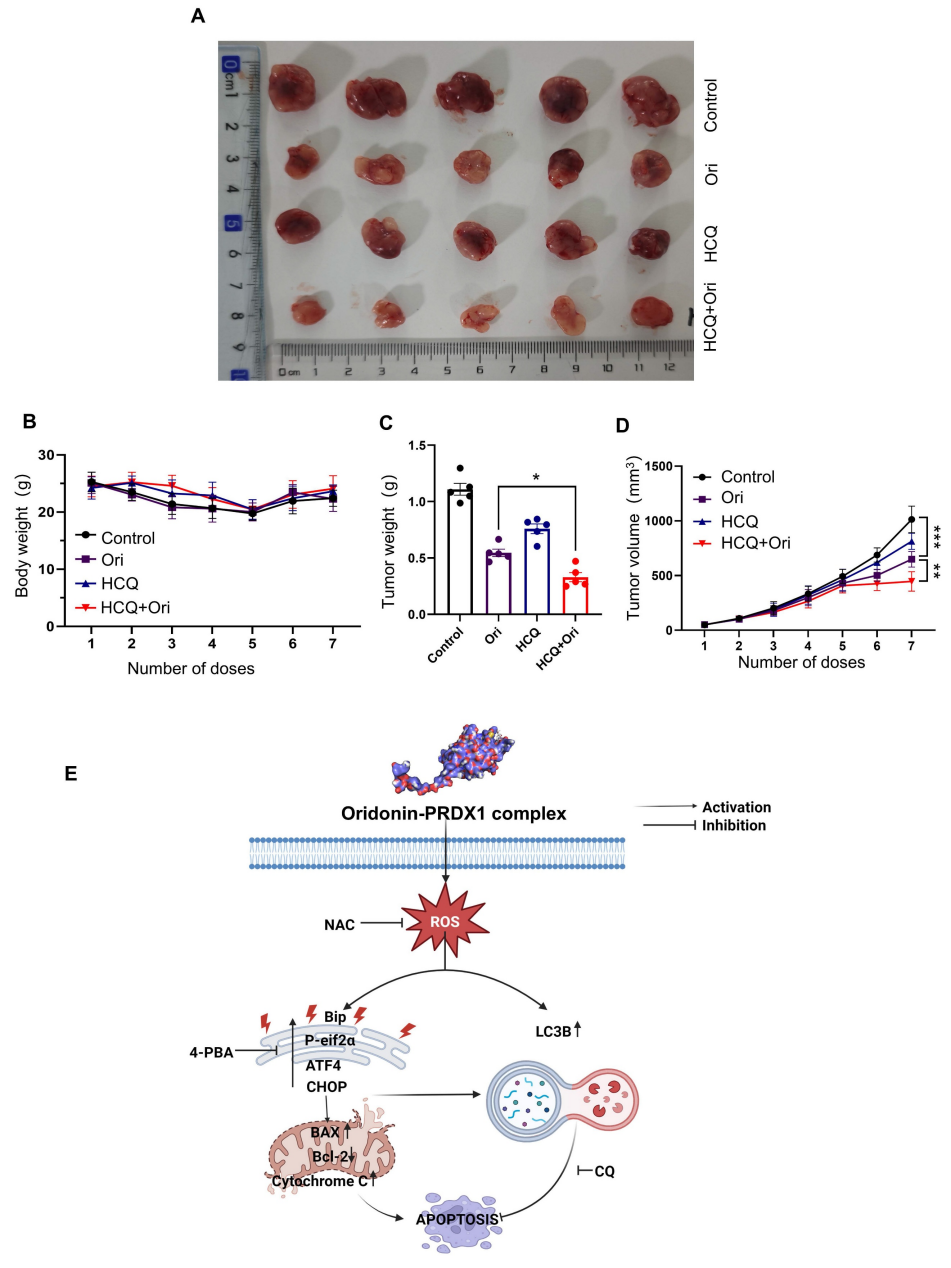
**The anti-renal cancer effects of oridonin and its mechanisms.** (A) Schematic diagram of tumor anatomy. (B) Graph of dynamic monitoring of mouse body weight (n=5). (C) Graph of statistical analysis of tumor weight. (D) Tumor volume growth curve. The presented data are expressed as mean ± SEM (n=5), *P < 0.05, **P < 0.01, ***P < 0.001. (E) Illustrative schematic of the putative mechanism underlying Oridonin-mediated apoptosis in RCC Cells. Oridonin induces cytotoxicity in RCC (786-O) cells. Specifically, Oridonin engages in direct interaction with PRDX1 via covalent binding, ultimately resulting in the accumulation of ROS. This ROS accumulation further exacerbates apoptosis by activating the ER stress pathway. Notably, ER stress inhibitor and ROS scavenger NAC attenuate oridonin-induced apoptosis. Importantly, both of the Oridonin-induced ROS and the ER stress trigger cytoprotective autophagy.

In order to comprehensively assess the anti-tumor potential of Oridonin, further systematic* in vivo* experiments were conducted using an OSRC-2 cell-derived xenograft mouse model. Since our *in vitro* findings indicated that autophagy inhibitors increase Oridonin-induced cell death, we also tested whether combining Oridonin with the autophagy inhibitor HCQ would be more effective against tumors in an animal model of RCC. HCQ is a well-known lysosomotropic agent that accumulates in lysosomes and increases their internal pH, thereby impairing lysosomal hydrolase activity and disrupting autophagosome-lysosome fusion 27. Treatment with Oridonin with HCQ was initiated when the average size of subcutaneous tumors in mice reached a certain size. The results showed that Oridonin significantly inhibited tumor growth while preserving their body weight compared to the drug control (**Fig. 7A-D**). Moreover, H&E staining showing that Oridonin treatment did not induce obvious toxicity in the liver and kidney (**Fig. S5**). Importantly, combining of Oridonin with HCQ further inhibited tumor growth, suggesting that autophagy inhibitor enhances the anti-tumor efficacy of Oridonin.

## Discussion

In this study, we report for the first time, to our knowledge, the direct covalent binding of Oridonin to PRDX1, resulting in the accumulation of ROS in renal cancer cells. The accumulation of ROS subsequently initiates apoptosis by activating ER stress. Remarkably, in response to Oridonin exposure, the ER stress induced by ROS also elicits cytoprotective autophagy (**Fig. 7E**). Our findings reveal a novel protein target for Oridonin, providing fresh insights into its anti-cancer mechanisms.

In recent times, several key natural compounds, such as andrographolide, and artemisinin 28, 29, have been recognized for their direct protein targets. The present study suggests that Oridonin specifically targets PRDX1 proteins by binding to their cysteine residues, thereby inhibiting their antioxidant activity. The PRDX family has emerged as a focal point in antitumor research, utilizing thioredoxin as an electron donor to mitigate ROS accumulation 30. Among the PRDX proteins, PRDX1 stands out due to its extensive study and high expression in multiple tumor types. One of the key antioxidant functions of the PRDX family is converting H_2_O_2_ to H_2_O and O_2_, safeguarding cells from apoptosis caused by elevated H_2_O_2_ levels. Here, we focused on targeting PRDX1 in RCC based on evidence showing its significant overexpression in RCC cancer cells compared to normal renal tissues. Critically, high PRDX1 expression strongly correlates with poor patient prognosis, including higher tumor stage and grade, increased metastasis risk, and shorter overall survival 31. This association underscores a potent pro-oncogenic role for PRDX1 in RCC. As such, highly overexpressed of PRDX1 link to aggressive disease in RCC provides a compelling disease-specific rationale for therapeutic targeting, distinguishing it as a highly promising strategy for this malignancy. However, RCC research in this context is relatively scant, and our study contributes to filling this gap. Therefore, we postulate that Oridonin-induced ROS accumulation through PRDX1 inactivation may represent a potential mechanism that may underlie its anticancer properties across different cancer types, including renal cancer. Moreover, we showed that Oridonin exhibits significant selective toxicity toward cancer cells while having minimal impact on normal kidney cells. Its selective mechanism primarily manifests the higher expression of redox status and higher expression of PRDX1 in cancer cells. Here, we showed that JNK activation is a key downstream target for oridonin-induced cell death by targeting PRDX1. Previous studies have well-established that ASK1 (Apoptosis signal-regulating kinase 1) is a core kinase linking oxidative stress (ROS accumulation) to JNK activation. Of particular importance, PRDX1 has been shown to be a key physiological inhibitor of ASK1^32^. At steady state, PRDX1 maintains ASK1 in an inactive state by forming a complex with ASK1 32. It is most likely that when Oridonin specifically covalently modifies the Cys173 site of PRDX1, this critical modification event directly results in the loss of the peroxidase activity of PRDX1 and likely triggers a change in its protein conformation. This functional inhibition and structural perturbation destabilize the complex formed between PRDX1 and ASK1, resulting in the release of ASK1 from its intrinsic inhibitory state. The free ASK1 is then activated by autophosphorylation. Activated ASK1 then phosphorylates and activates its immediate downstream kinases MKK4 and/or MKK7 (MAP2K) to further activates JNK. Although the focus of this study was on PRDX1 and JNK, the activation of ASK1 may be a key link in this chain, and future studies could directly examine the activation status of ASK1 to further confirm this possibility.

Despite Oridonin's promising anticancer effects by targeting PRDX1, potentially across various cancer types, its clinical application faces challenges, including moderate potency, solubility, and bioavailability issues 33. Firstly, the ubiquitous expression of PRDX1 raises concerns about off-target effects and systemic toxicity, especially given its physiological role in maintaining redox homeostasis in normal tissues. Therefore, developing highly selective inhibitors or modulators that preferentially affect PRDX1 in tumor cells is critical. Indeed, recent studies have developed novel chemical syntheses to produce nitrogen-rich Oridonin analogs based on structurally diverse diterpenoids, aiming to overcome these limitations and create more effective derivatives against different cancers 5. The medicinal chemistry advancements in Oridonin research facilitate the development of new derivatives targeting a range of proteins 34. Secondly, effective drug delivery remains a significant hurdle. This compound is highly hydrophobic, resulting in poor solubility and bioavailability, which limits its absorption and systemic exposure. To address this issue, developing novel formulations such as liposomes, nanoparticles, or cyclodextrin complexes is an effective strategy to enhance solubility and prolong circulation time. For example, recent studies have shown that PEGylated Oridonin nanoparticles can significantly improve drug delivery to tumor sites 35. ER stress, characterized by the malfunction of the ER, prompts cells to activate a series of signaling pathways as a defensive response to this distress. Moderate ER stress has been shown to stimulate various oncogenic processes, including cancer cell proliferation, metastasis, resistance to chemotherapy, blood vessel formation and immune system evasion 25. Conversely, severe ER stress, arising from the unchecked accumulation of misfolded proteins in organelles, initiates a terminal unfolded protein response (UPR), ultimately resulting in cellular apoptosis. Previous studies have shown that Oridonin can induce ER stress in a range of tumor cell types, including colorectal 36 and laryngeal cancers 37. This induction is achieved through the upregulation of ER stress-related proteins such as P-PERK, GRP-78, P-EIF2α, ATF4, and CHOP. These alterations in protein expression subsequently led to apoptosis by causing morphological changes in the ER, such as an increase in vesicular number and irregular shapes. In this research endeavor, we examined the impact of Oridonin on ER stress within renal cancer cells (786-O and OSRC-2). Our findings were consistent with previous research, showing that Oridonin increased the levels of ER stress-related proteins in these cells. Notably, the administration of NAC and ER stress inhibitors was found to alleviate this ER stress state, suggesting potential therapeutic applications in the management of renal cancer.

Autophagy is an intracellular self-degradation process and is implicated in multiple disease 38, 39, and in the case of cancer, the role of autophagy is twofold. In certain instances, autophagy acts as a barrier to cancer progression; however, recent studies have demonstrated that enhanced autophagic flux, induced by autophagy, can facilitate the proliferation of tumor cells 40. Either induction or inhibition of autophagy may be a new strategy to enhance the efficacy of anticancer drugs. Some studies have shown that chemotherapeutic resistance of certain cancers can be overcome and therapeutic efficacy can be improved by inhibiting autophagy. However, it has also been shown that in some cases, induction of autophagy can promote cancer cell death and thus exert therapeutic effects. Therefore, a crucial inquiry in cancer treatment revolves around whether autophagy triggers the demise of cancer cells or shields them from destruction. Previous studies have shown that Oridonin can induce autophagy in Human Cervical Carcinoma HeLa Cells 6, Pancreatic Cancer Cells 41 and Small Cell Lung Cancer Cells 42. However, there exist contrasting findings indicating that Oridonin exerts an inhibitory impact on autophagy within synoviocytes 43, which is likely attributable to both cell-type-specific and context-dependent factors due to metabolic state, and stress signaling pathways. Our study has shown that by inhibiting autophagy, the antitumor efficacy of Oridonin was potentiated. Therefore, autophagy had a protective effect on RCC cell in this study.

While SPR is a standard method for confirming direct molecular interactions, our attempts to detect binding between Oridonin and PRDX1 using SPR were inconclusive, likely due to technical challenges such as PRDX1 immobilization or Oridonin solubility issues. To address this, we employed complementary approaches. We identified Oridonin covalent binds to Cys173 of recombinant PRDX1. CETSA-WB revealed increased thermal stability of PRDX1 upon Oridonin treatment, suggesting target engagement in cells. Additionally, Oridonin inhibited PRDX1 enzymatic activity and induced phenotypes-ROS accumulation, cell death, and autophagy-that were modulated by PRDX1 overexpression or knockdown. These findings support PRDX1 as a direct and functionally relevant target of Oridonin, despite the absence of definitive SPR data.

Crucially, knocking down PRDX1 alone fails to replicate the full spectrum of Oridonin's pharmacological activity. Our findings, establishing PRDX1 as Oridonin's direct and critical target, nevertheless do not preclude the compound's simultaneous engagement of other proteins 44, consistent with existing literature. Such additional targets may act synergistically with PRDX1 inhibition or via distinct pathways, culminating in the profound ROS surge and cell death characteristic of Oridonin treatment. Thus, PRDX1 represents a key hub within Oridonin's complex multi-target network, not the only player. Future studies of pursuing additional potential targets of Oridonin and elucidating synergistic partners will be essential for fully resolving its mechanism and designing effective combination therapies for cancers.

In summary, our findings demonstrate that Oridonin effectively inhibits RCC growth in vitro and reveal a novel target for the natural small-molecule compound Oridonin. Consequently, Oridonin can serve as a natural lead compound for developing PRDX1-targeted, RCC-selective therapies either alone or in combination with autophagy inhibitors for cancer treatment. Furthermore, by identifying a new target for Oridonin and demonstrating its therapeutic efficacy in RCC, this study provides a unique strategy for discovering and validating natural product targets in cancer therapy.

## Supplementary Material

Supplementary figures.

## Data Availability

All data supporting the findings of this study are available either within the article, or from the corresponding author upon reasonable request.
