# Supplementary material for: Oridonin targets PRDX1 to promote apoptosis by inducing ROS-mediated ER stress and modulating autophagy
Source: Int J Biol Sci. 2026 Jun 17;22(12):6363–80. doi: 10.7150/ijbs.110208 (PMC13412092; doi:10.7150/ijbs.110208)
Supplement: Supplementary file 1 — Supplementary figures. [file ijbsv22p6363s1.pdf]

Supplementary Figures

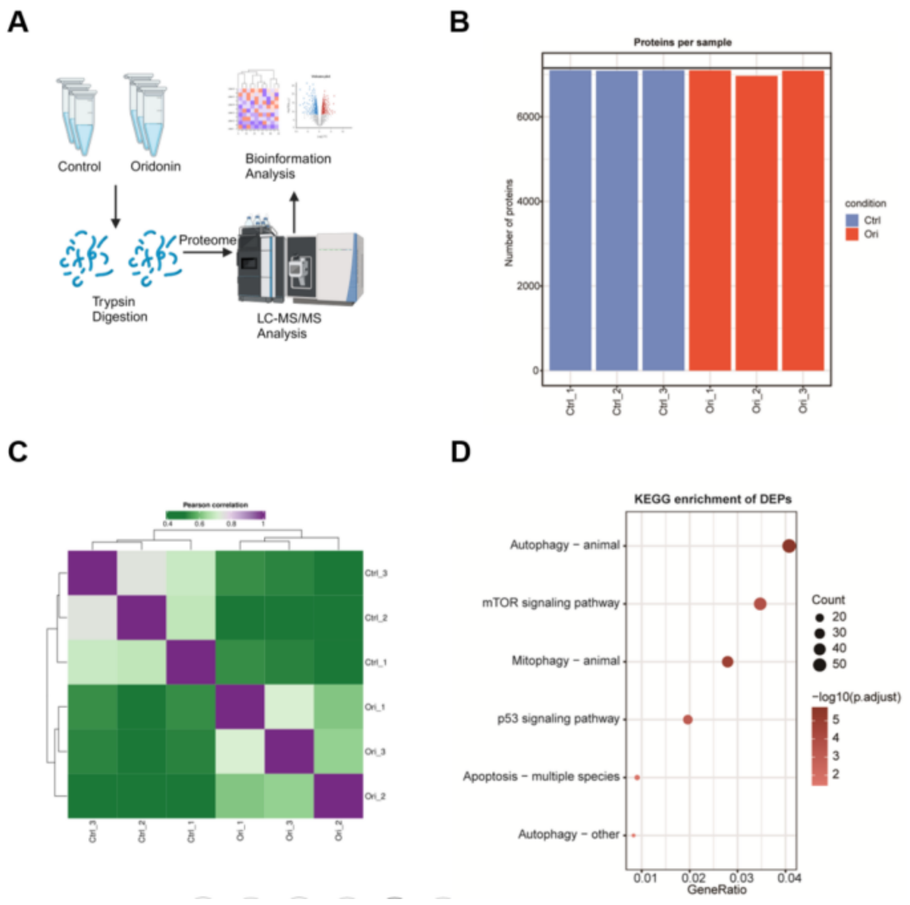

Fig S1. (A) The schematic workflow of proteomic analysis. (This image was created by BioRender.com). **(B)**. The amount of protein detected in each sample of treatment group and control group was uniform, and the detection abundance was fluctuating at 6500. **C**. Heatmap of the DEGs among different samples. **(D)** DEGs related to KEGG pathways after treatment of cells with Oridonin.

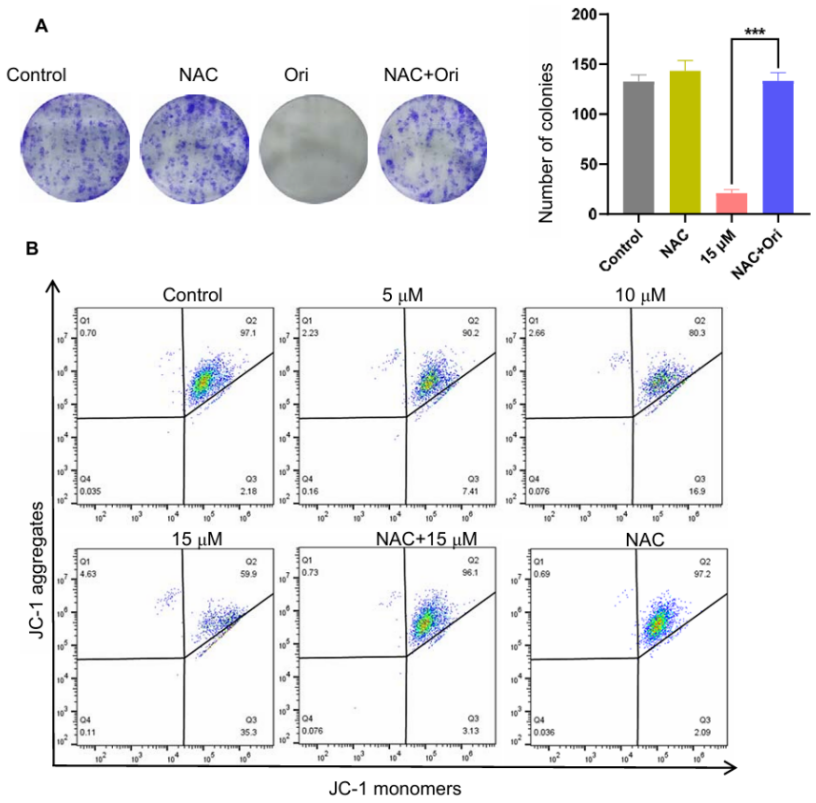

Fig S2. (A) The colony formation of 786-O cells was counted after cells were treated with Oridonin and NAC for 7 days and the colony formation ratio was quantified. **(B)**. The MMP was measured with the fluorescent mitochondrial probe JC-1 and assessed by flow cytometry.

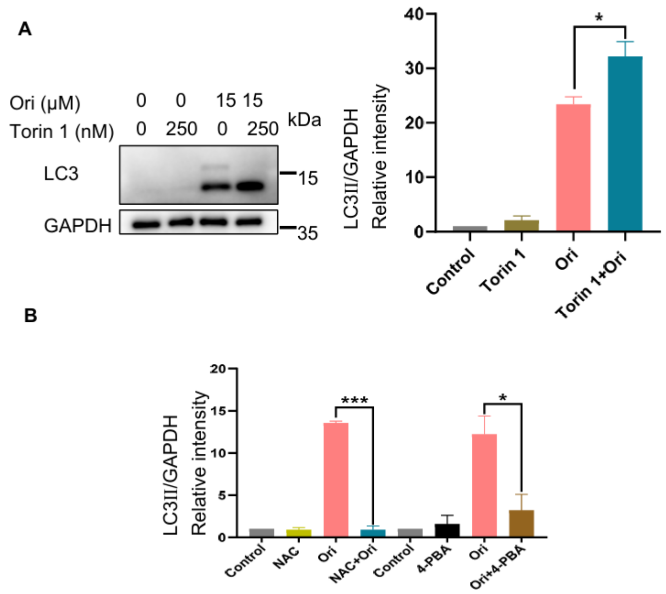

Fig S3. **(A)** The expression of autophagy markers LC3-II was detected by western blotting after Totin 1(250 nM) and Oridonin were used to treat 786-O cells for 24 h. **(B)**4-PBA (1 mM) was used alone or in combination with Oridonin to treat cells for 24 h, The expression levels of LC3-II were quantified.

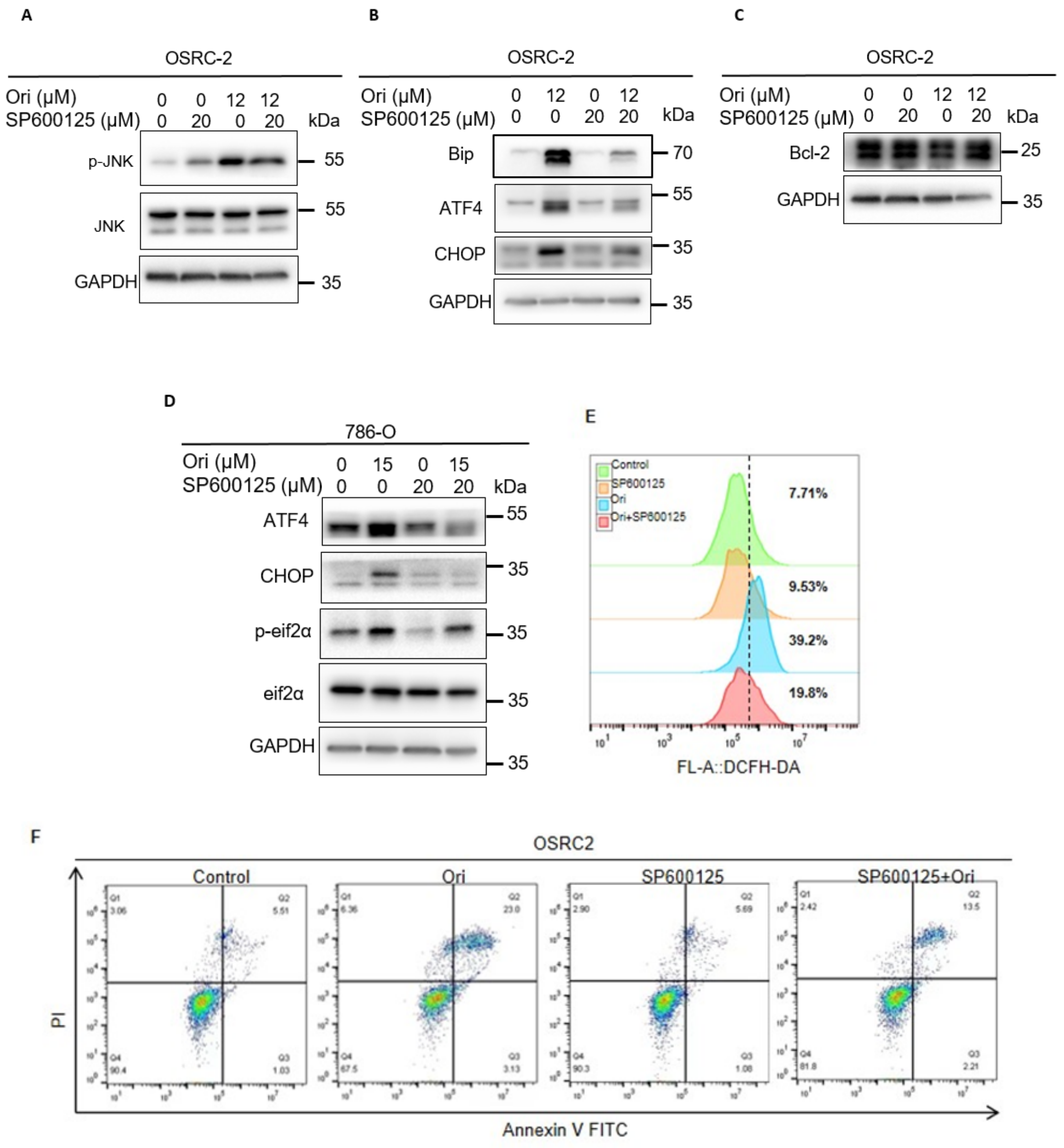

Fig S4. **(A-C)** After pretreatment with SP600125 (20 μM) for 2 h, Western blotting was performed to analyze the effect of Oridonin on the expression levels of P-JNK and JNK **(A),** ER stress markers Bip, ATF4, and CHOP **(B),** as well as Bcl-2 protein expression levels in OSRC-2 cells **(C). (D)** The effect of Oridonin on the expression levels of ER stress markers Bip, ATF4, CHOP, p-eIF2α, and eIF2α proteins in 786-O cells was analyzed by Western blotting after pretreatment with SP600125 (20 μM) for 2 h. **(E)** After pretreatment with JNK inhibitor SP600125 (20 μM) for 2 h, ROS levels in OSRC-2 cells were assessed by Oridonin using DCFH-DA staining, followed by flow cytometry analysis. **(F)** Pretreatment with the JNK inhibitor SP600125 (20 μM) for 2 h attenuated Oridonin-induced apoptosis. The occurrence of apoptosis was determined by Annexin V-FITC/PI staining and flow cytometry analysis.

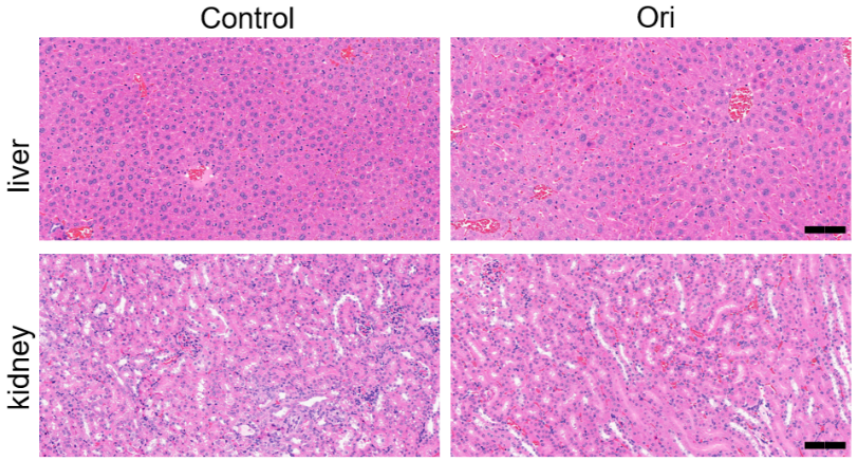

Fig S5. H&E staining showing that Oridonin treatment did not induce obvious toxicity (Scale bar: 50 μM).
